# Alterations in Cerebellar Functional Connectivity Are Correlated With Decreased Psychomotor Vigilance Following Total Sleep Deprivation

**DOI:** 10.3389/fnins.2019.00134

**Published:** 2019-02-21

**Authors:** Ying Zhang, Yebing Yang, Yan Yang, Jiyuan Li, Wei Xin, Yue Huang, Yongcong Shao, Xi Zhang

**Affiliations:** ^1^Department of Neurology, The Second Medical Center, Sleep Medicine Research Center, National Clinical Research Center for Geriatric Disease, Chinese PLA General Hospital, Chinese PLA Medical School, Beijing, China; ^2^Department of Psychology Medical, The Eighth Medical Center, Chinese PLA General Hospital, Beijing, China; ^3^The Sixth Medical Center, Chinese PLA General Hospital, Beijing, China; ^4^Department of Radiology, The Eighth Medical Center, Chinese PLA General Hospital, Beijing, China; ^5^Department of Magnetic Resonance Imaging, Beijing Shijitan Hospital, Capital Medical University, Beijing, China; ^6^Army Medical University, Chongqing, China; ^7^School of Psychology, Beijing Sport University, Beijing, China

**Keywords:** sleep deprivation, functional connectivity, cerebellum, fMRI, psychomotor vigilance

## Abstract

Previous studies have reported significant changes in functional connectivity among various brain networks following sleep restriction. The cerebellum plays an important role in information processing for motor control and provides this information to higher-order networks. However, little is known regarding how sleep deprivation influences functional connectivity between the cerebellum and the cerebral cortex in humans. The present study aimed to investigate the changes in cerebellar functional connectivity induced by sleep deprivation, and their relationship with psychomotor vigilance. A total of 52 healthy men underwent resting-state functional magnetic resonance imaging before and after 36 h of total sleep deprivation. Functional connectivity was evaluated using region of interest (ROI)-to-ROI analyses, using 26 cerebellar ROIs as seed regions. Psychomotor vigilance was assessed using the psychomotor vigilance test (PVT). Decreased functional connectivity was observed between cerebellar seed regions and the bilateral postcentral, left inferior frontal, left superior medial frontal, and right middle temporal gyri. In contrast, increased functional connectivity was observed between the cerebellum and the bilateral caudate. Furthermore, decrease in functional connectivity between the cerebellum and the postcentral gyrus was negatively correlated with increase in PVT reaction times, while increase in functional connectivity between the cerebellum and the bilateral caudate was positively correlated with increase in PVT reaction times. These results imply that altered cerebellar functional connectivity is associated with impairment in psychomotor vigilance induced by sleep deprivation.

## Introduction

Sleep deprivation is very common in today’s society, and most young people have experienced some degree of acute or chronic sleep loss due to continuous or shift work (Drake et al., 2004). Moreover, previous studies have demonstrated that at least 30% people sleep less than 7 h/d (Jones et al., 2008), and that this phenomenon is more prominent among older adults (Chatzitheochari and Arber, 2009). Sleep deprivation is also common among patients with sleep disorders, making sleep an international health concern (Robotham, 2011).

Acute sleep deprivation can severely affect cognitive function in humans: decrease in levels of attention and working memory is observed after 24 h of total sleep deprivation (TSD) (Zhou et al., 2017). After 36 h of continuous resting wakefulness, decrease in psychomotor vigilance is observed (Lim and Dinges, 2010; Nakashima et al., 2017), along with significant impairments in the operational process. Animal studies have revealed that, in addition to cognitive impairment, lack of sleep also causes disruptions in immune function (Mullington, 2009). These alterations in bodily homeostasis produce significant changes in behavior, including balance impairments, decreased attention, and reduced decision-making capacity, which may lead to mistakes or accidents at work (Drummond et al., 1999; Zhou et al., 2017).

Sleep deprivation severely damages a human’s ability to respond to stimuli in a timely fashion (Zhou et al., 2017). These deficiencies are largely attributed to the failure of vigilant attention, which many theorists believe constitutes the cornerstone of more complex cognitive processes (Lim and Dinges, 2010). One of the main paradigms utilized to examine vigilant attention is the psychomotor vigilance test (PVT), a high-signal reaction time test that is extremely sensitive to sleep deprivation (Dongen and Dinges, 2005; Robert and Eickhoff, 2013). Previous research has indicated that the PVT activates not only the basal ganglia and sensorimotor cortex, but also the right frontal-parietal attention network (FPN). Optimal performance during the PVT appears to depend on the activation of the sustained attention and motor systems. Poor performance after TSD may be due to disengagement from tasks and related inattention, which are related to changes in activation of the midline structures involved in the brain’s default mode network (DMN) (Drummond et al., 2005).

Previous studies have reported that cognitive decline after sleep deprivation is associated with imbalances in functional brain networks such as the DMN, FPN, dorsal attention network (DAN), and salience network (SN) (Wirsich et al., 2017). Indeed, prolonged resting wakefulness causes progressive worsening of the imbalances in the functional relationships among these brain networks. The SN is involved in detecting, integrating, filtering external versus internal stimuli, and allocating attention (Seeley et al., 2007; Menon, 2011). After TSD, a significant increase in SN-DMN coupling shows the upregulated assignment of saliency to internal mental events for survival, while the SN-FPN coupling also increases slowly to meet the demands of the longer waking state. The biased resource allocation toward the DMN indicates that the homeostatic process for survival was prioritized during sleep deprivation. Fluctuations in sustained attention are related to the recruitment of the DAN, which consists of the frontal eye fields and inferior parietal sulcus (Fox et al., 2005). Functional neuroimaging has identified brain areas that respond with an increase in activation to goal-oriented cognitive tasks (Gazes et al., 2012). In addition, the functional link within the DAN decreases after TSD (Zhou et al., 2017). Dysfunction in these brain networks may be the foundation of profound changes in cognition and behavior during and after sleep deprivation (Krause et al., 2017).

The cerebellum plays an important role in information processing for the maintenance of balance (Keren-Happuch et al., 2014; Noroozian, 2014). Studies have shown that the cerebellum consists of several modules, each of which is relatively independent and connected to specific areas of the cerebral cortex (Houk and Wise, 1995). The cerebellum plays an important role in the control and coordination of sensorimotor function, such as motor speech control, motor sensory synchronization, cortical motor excitability control, and motor-related sensory data acquisition control. In addition, there is growing evidence that the cerebellum not only contributes to motor control, but also improves “cognitive” function (Ramnani, 2006; Manto et al., 2012).

Based on recent empirical studies, attention to non-cortical-mediated basal ganglia-cerebellum interactions may fundamentally change our view on how these subcortical regions interact with each other and with the cortex to regulate motor and non-motor behaviors (Middleton and Strick, 2000; Caligiore et al., 2017). Dysfunction of cortico-cerebellar-thalamo-cortical circuit can explain various behavioral symptoms of schizophrenia, Parkinson’s disease, and Tourette syndrome (Houk and Wise, 1995; Andreasen et al., 1998, 1999; Caligiore et al., 2017).

Sleep deprivation is known to decrease voluntary motor control of behavioral performance, which may be associated with decrease in the activity and/or functional connectivity of the cerebellar network (Wang et al., 2014). Recent studies have revealed that the cerebellum plays an important role in the control of sensation and movement (Manto et al., 2012; Overwalle and Mariën, 2016; Caligiore et al., 2017). However, few studies have investigated the functional cerebellar network following sleep deprivation. Thus, the role of the cerebellum and its associated networks in the psychomotor vigilance changes caused by sleep deprivation remains unknown. The present study aimed to investigate alterations of the cerebellar functional connectivity before and after sleep deprivation, and to determine the association between the cerebellar functional connectivity changes and alterations in indicators of PVT.

## Materials and Methods

### Participants

This study recruited 52 healthy, right-handed adult men (age: range, 18–29 years; mean ± SD, 23.54 ± 2.82 years) from Beijing Normal University and Beihang University as paid volunteers using advertisements. None of the included participants had previously participated in psycho-physiological experiments, and all had normal or corrected-to-normal vision. The exclusion criteria were as follows: diseases of the central and peripheral nervous systems, head trauma, cardiovascular diseases and/or hypertension, cataracts and/or glaucoma, pulmonary issues, and alcohol or drug abuse. None of the participants exhibited evidence of clinical symptoms, as assessed using the Symptom Checklist-90, Self-rating Anxiety Scale, or Self-rating Depression Scale (Derogatis et al., 1976). In addition, all participants obtained intelligence scores within the normal range (Raven’s Progressive Matrices Test, intelligent quotient > 100), and were without sleep disorders (Pittsburgh Sleep Quality Index < 7). All participants were requested to maintain a regular sleep schedule and to refrain from napping or consuming alcohol, caffeine, or chocolate for 1 week prior to and throughout the study. All participants had established regular sleep patterns (approximately 8 h of sleep per night). This study was approved by the Research Ethics Committee of the General Hospital of People’s Liberation Army and Beihang University (Beijing, China). Written informed consent was obtained from each participant after a complete description of the study had been provided.

### Behavioral Measures

The Visual Analogue Scale, a Likert-type rating scale (0–10), was employed to assess the alertness level of participants prior to and after TSD (Lee et al., 1991). We selected PVT to test subjects’ psychomotor vigilance level. In this text, red dots (diameter, 3 cm; viewing angle, 1.5 × 1.5°) were displayed in the center of the white background on the liquid crystal display screen with 1024 × 768-pixel resolution (refresh rate, 60 Hz). At the beginning of each trial, subjects focused on a “+” symbol at the center of the screen for 400 ms. After that, the red dots appeared for 1000 ms and disappeared once the subjects responded. The interval between trials was 4000–6000 ms (mean, 5000 ms). The stimulus interval was pseudo-randomized. Subjects were instructed to respond as soon as possible after the stimulus appeared, but early response was not allowed. Participants completed the PVT with 120 trials (Basner and Dinges, 2005).

### Experimental Paradigm

Experiments were conducted at Institute of Beihang University (Beijing, China), with the nursing staff present at all times. Every two participants were teamed together and monitored by operators throughout the experiment to ensure that they remained awake. Participants were not allowed to leave the laboratory during the TSD period until they were escorted to the functional magnetic resonance imaging (fMRI) facility.

**FIGURE 1:**
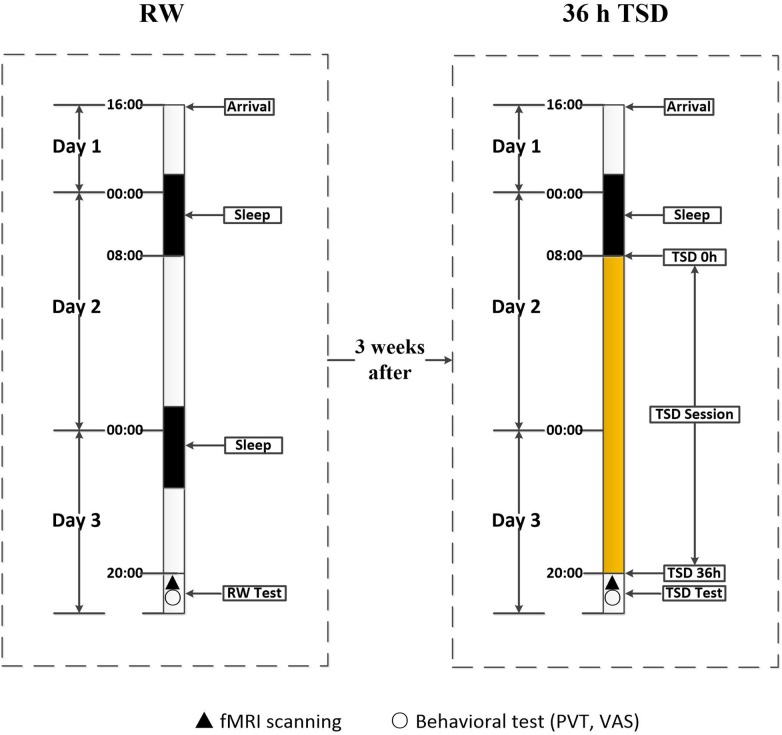
Experimental design and protocol. The experiment consisted of an RW and a TSD component, which were separated by an interval of 3 weeks. During each component, participants entered the laboratory at 4:00 PM on the first day, and underwent functional magnetic resonance imaging and behavioral testing at approximately 8:00 PM on the third day. In the RW component, participants went through two routine nocturnal sleep periods. In the TSD component, participants underwent 36 h of TSD after one routine nocturnal sleep period. The 36 h of TSD started at 8:00 AM on the second day and ended at 8:00 PM on the third day. TSD, total sleep deprivation; RW, rested wakefulness.

Participants underwent scanning twice: once during rested wakefulness (RW) and once after 36 h of TSD. The 36 h of TSD started at 8:00 AM on the second day and ended at 8:00 PM on the third day. The two scanning sessions were conducted 3 weeks apart to minimize the possibility of residual TSD side effects among participants who had undergone the TSD scan prior to the RW scan. After each scan session, the PVT was used to measure the psychomotor vigilance of each participant. The self-rated Visual Analogue Scale was used to evaluate subjective alertness. Both scan sessions were performed at the same time (8:00 PM), and the scanning order was counter-balanced across participants to reduce the potential influence of scan order (Figure 1).

### Resting-State Paradigm

Participants underwent structural MRI and fMRI that included resting-state procedures. Participants were positioned in the scanner with their heads comfortably restrained to reduce head movement. During the resting-state scans, participants were instructed to keep their eyes open, remain as motionless as possible, and not to think of anything in particular. The resting-state scan lasted for 380 s. The investigators were able to observe the participant’s eyes, to confirm that they remained open, using a camera throughout the experiment. Before each session, participants were reminded to keep awake using a microphone. After each session, they were asked whether they were awake in the previous session, and all the participants confirmed that they were awake. A pulse oximeter was attached to the participant’s finger to record cardiac activity. In addition, participants wore a pressure belt around the abdomen to record respiratory activity. The cardiac and respiratory signals were collected and synchronized to the fMRI data to ensure that physiologic variations could be removed during the regression analysis.

### Data Acquisition and Processing

Standard resting-state functional images and T2^∗^-weighted echo-planar images were obtained using 3T MRI scanners. The SPM 12 software (University College London^1^) and CONN toolbox version 17a (Neuroimaging Informatics Tools and Resources Clearinghouse^2^) were employed for functional image preprocessing (Whitfield-gabrieli and Nieto-castanon, 2012). The first 10 volumes of the functional time series for each epoch were discarded for signal stabilization, and for participants to become accustomed to scanning noise. The data volumes were then corrected by registration to the first volume to account for head motion. Rotational and translational movements of all participants were within 2 mm or 2°, respectively, in the *x*, *y*, and *z* planes. The volumes were normalized to the standard echo planar imaging template in the Montreal Neurological Institute space and resliced to 3 mm × 3 mm × 3 mm. The resulting images were spatially smoothed using a Gaussian filter and a 6-mm full-width at half-maximum kernel. Subsequently, the data were temporally filtered using a band-pass filter of 0.009–0.1 Hz. Linear trend elimination was followed by removal of data via multiple regression analysis, which included averaging of signals from white matter, cerebrospinal fluid, and the whole brain, as well as parameters obtained from the six head motion directions. This regression procedure was used to reduce the influence of spurious variance on neuronal activity.

### Functional Connectivity Analysis

CONN is a MATLAB (MathWorks, Inc., Natick, MA, United States)-based cross-platform software used to compute and analyze the functional connectivity of brain regions based on fMRI signals (Whitfield-gabrieli and Nieto-castanon, 2012). We utilized the CONN toolbox implemented in the 2016 version of MATLAB and SPM12 to process resting-state fMRI scans into region-of-interest (ROI)-to-ROI connectivity matrices, test hypotheses, and visualize data (Whitfield-gabrieli and Nieto-castanon, 2012). The CONN toolbox was used to create participant-specific ROI files for 116 ROIs, including 26 cerebellar ROIs, and register them to the participant space. All ROIs were drawn from the Automated Anatomical Labeling atlas (Tzourio-Mazoyer et al., 2002). These bilateral ROIs were previously defined in detail by Tzourio-Mazoyer et al. (2002). In order to minimize the impact of motion and physiological noise factors, the CompCor function was used for spatial and temporal preprocessing to define and remove confounds in the blood-oxygen-level dependent signal (Behzadi et al., 2007). Fisher transformation was then used to convert the correlation coefficients to normally distributed scores, in order to allow second-level general linear model analysis (Whitfield-gabrieli and Nieto-castanon, 2012). The correlation maps were dependent on the specific location of the seed regions so that functionally and anatomically heterogeneous ROIs were dissociated, enabling us to delineate functional anatomy in the brain based on sharp transitions in correlation patterns, which signal functional boundaries across the cortex (Whitfield-gabrieli and Nieto-castanon, 2012).

Functional connectivity measures were computed between seed regions for ROI-to-ROI analysis and to identify patterns of ROI-to-ROI connectivity. The CONN toolbox was used to obtain a linear measure of functional connectivity based on bivariate correlation and regression coefficients, as well as their associated multivariate measures of semi-partial correlation and multivariate regression coefficients (Whitfield-gabrieli and Nieto-castanon, 2012). Wilks’s lambda or F-statistics were used to evaluate ROI-to-ROI connectivity matrices for each participant depending on the dimensionality of the within- and between-subjects’ contrasts. Effect sizes for connectivity contrasts between all ROI sources were calculated alongside *T*, *F*, and *X* values; and false discovery rate-corrected *p*-values were calculated for each specified second-level analysis. The *F*-test was used to calculate the multivariate connectivity strength for each seed-level threshold.

We compared functional connectivity between the RW and TSD scans using two-tailed paired *t*-tests. The analysis of functional connectivity between TSD and RW states was performed with the *p*-value of 0.025 (false discovery rate-corrected) for ROI-to-ROI tests (Whitfield-gabrieli and Nieto-castanon, 2012).

### Behavioral Correlations

We conducted a correlation analysis to investigate the relationship between changes in functional connectivity and psychomotor vigilance before and after sleep deprivation. We calculated the Pearson correlation coefficients between the changes in metrics of PVT and functional connectivity before and after sleep deprivation. The level of statistical significance was set at *p* < 0.05.

## Results

### Physiological Data

**FIGURE 2:**
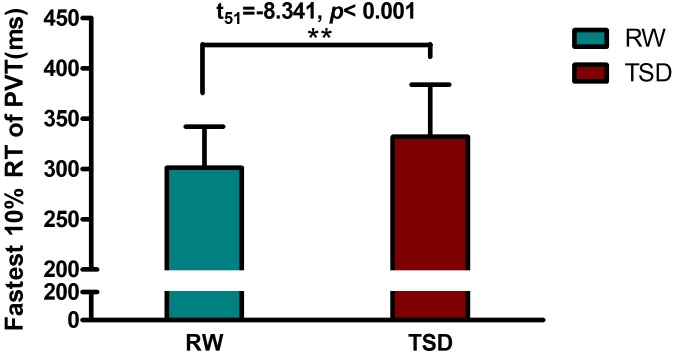
Increased fastest 10% reaction times of PVT between the RW and TSD conditions. The error bars represent the standard deviation from the mean; ^∗∗^*p* < 0.01 paired *t*-test. PVT, psychomotor vigilance test; TSD, total sleep deprivation; RW, rested wakefulness; fastest 10% reaction times, the mean of the 10% fastest trials for the PVT.

Demographic data, psychological traits, and sleep characteristics are shown in Table 1. Paired *t*-tests revealed significant differences between the RW and TSD conditions in alertness (*t* = 8.339, *p* < 0.001), fastest 10% reaction times of PVT (*t* = -8.203, *p* < 0.001) (Figure 2), mean reaction times (*t* = -8.701, *p* < 0.001), and lapse probability (*t* = -3.890, *p* < 0.001) (Table 1).

**Table 1:** Demographic data, psychological traits, and sleep characteristics (*n* = 52).

	RW state		TSD state	*t*	*p*-value
Age (years)	23.54 ± 2.82	–	–	–	–
Men (n[%])	52 (100%)	–	–	–	–
BMI (kg/m^2^)	22.84 ± 1.90	–	–	–	–
Education (years)	16.08 ± 1.38	–	–	–	–
SAS	37.40 ± 6.99	–	–	–	–
SDS	37.62 ± 7.13	–	–	–	–
PSQI	3.29 ± 1.27	–	–	–	–
SCL-90	127.37 ± 27.91	–	–	–	–
IQ	113.35 ± 7.13	–	–	–	–
Alertness (VAS)^a^	–	8.19 ± 1.48	5.31 ± 2.61	8.339	<0.001
Fastest 10% RT (PVT)^b^	–	301.23 ± 40.79	331.93 ± 51.89	-8.341	<0.001
Mean RT (PVT)	–	394.69 ± 51.03	435.48 ± 43.29	-8.701	<0.001
Lapse probability (PVT)^c^	–	13.56% ± 10.64%	20.23% ± 15.15%	-3.890	<0.001


*RW, rested wakefulness; TSD, total sleep deprivation; BMI, body mass index; SAS, Self-rating Anxiety Scale; SDS, Self-rating Depression Scale; PSQI, Pittsburgh Sleep Quality Index; SCL-90, Symptom Checklist-90; IQ, intelligence quotient; VAS, Visual Analogue Scale; RT, reaction time; PVT, psychomotor vigilance test*.

*^a^Alertness, assessed based on participant responses on the VAS (“alertness”)*.

*^b^Fastest 10% RT, calculated as the mean of the 10% fastest trials for the PVT*.

*^c^Lapse probability, calculated as the number of lapses (RTs ≥ 500 ms) divided by the number of valid stimuli*.

### Functional Connectivity Changes After TSD

**FIGURE 3:**
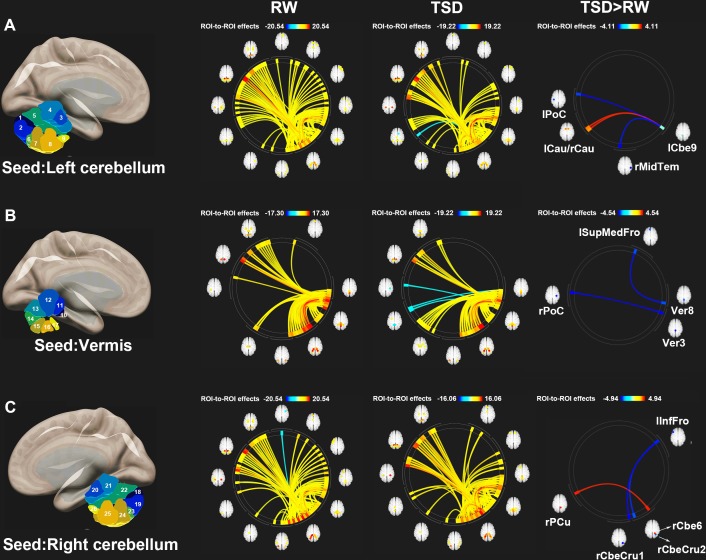
ROI-to-ROI functional connectivity of the cerebellum during the RW, TSD, and TSD > RW conditions. **(A)** Left cerebellum, **(B)** Vermis, and **(C)** Right cerebellum (*p* < 0.025), false discovery rate-corrected for ROI-to-ROI tests. ROI, region of interest; TSD, total sleep deprivation; RW, rested wakefulness; lCbe9, left cerebellum 9; lPoC, left postcentral gyrus; rMidTem, right middle temporal gyrus; rCau, right caudate nucleus; lCau, left caudate nucleus; Ver3, vermis 3; rPoC, right postcentral gyrus; Ver8, vermis 8; lSupMedFro, left superior medial frontal gyrus; rCbeCru1, right cerebellum crus 1; lInfFro, left inferior frontal gyrus; rCbeCru2, right cerebellum crus 2; rCbe6, right cerebellum 6; rPCu, right precuneus; 26 cerebellar ROIs: 1, left cerebellum crus 1; 2, left cerebellum crus 2; 3, left cerebellum 3; 4, left cerebellum 4–5; 5, left cerebellum 6; 6, left cerebellum 7b; 7, left cerebellum 8; 8, left cerebellum 9; 9, left cerebellum 10; 10, vermis 1–2; 11, vermis 3; 12, vermis 4–5; 13, vermis 6; 14, vermis 7; 15, vermis 8; 16, vermis 9; 17, vermis 10; 18, right cerebellum crus1; 19, right cerebellum crus 2; 20, right cerebellum 3; 21, right cerebellum 4–5; 22, right cerebellum 6; 23, right cerebellum 7b; 24, right cerebellum 8; 25, right cerebellum 9; 26, right cerebellum 10.

Figure 3 shows the ROI-to-ROI functional connectivity of the cerebellum for the RW, TSD, and TSD > RW conditions. After 36 h of TSD, participants exhibited reduced functional connectivity between the cerebellar seed regions and the bilateral postcentral, left inferior frontal, right middle temporal, and left superior medial frontal gyri. Increased functional connectivity was observed between the left cerebellum 9 and the bilateral caudate, between the right cerebellum 6 and the right precuneus (Figure 3). The effect sizes for functional connectivity between the cerebellum and whole-brain ROIs in the RW and TSD conditions are shown in Table 2.

**Table 2:** ROI-to-ROI functional connectivity statistics for an individual seed region: comparisons between RW and TSD scans (*t*-test).

Target region	AAL label	MNI center	*t*	FDR-corrected	*p*-values for
				*p*-value	correlations
lCbe9	Left cerebellum 9	(-9.96, -52.62, -51.13)			
lPoC	Left postcentral gyrus	(-43.08, -23.88, 48.07)	T(51) = -4.11	0.0167^a^	0.0330^b^
rMidTem	Right middle temporal gyrus	(55.4, -38.09, -2.27)	T(51) = -3.77	0.0217^a^	0.0538
rCau	Right caudate nucleus	(7.60, 13.38, 9.55)	T(51) = 3.60	0.0217^a^	0.0411^b^
lCau	Left caudate nucleus	(7.10, -13.26, 8.72)	T(51) = 3.58	0.0217^a^	0.0402^b^
Ver3	Vermis 3	(-1.42, -44.03, -15.52)			
rPoC	Right postcentral gyrus	(40.39, -23.46, 51.18)	T(51) = -4.54	0.0039^a^	0.0412^b^
Ver8	Vermis 8	(-1.44, -67.40, -38.72)			
lSupMedFro	Left superior medial frontal gyrus	(21.33, -6.83, 46.61)	T(51) = -4.31	0.0085^a^	0.7403
rCbeCru1	Right cerebellum crus 1	(32.19, -69.68, -35.22)			
lInfFro	Left inferior frontal gyrus	(-45.12, 27.50, 9.46)	T(51) = -4.94	0.0010^a^	0.3927
rCbeCru2	Right cerebellum crus 2	(11.86, -69.68, -35.22)			
lInfFro	Left inferior frontal gyrus	(-45.12, 27.50, 9.46)	T(51) = -4.23	0.0113^a^	0.8460
rCbe6	Right cerebellum 6	(-1.92, -69.33, -20.11)			
rPCu	Right precuneus	(24.23, 8.37, -55.26)	T(51) = 4.31	0.0086^a^	0.8706


*FDR, false discovery rate; ROI, region of interest; RW, rested wakefulness; TSD, total sleep deprivation, AAL: Automated Anatomical Labeling.*

*^*a*^*p* < 0.025; FDR-corrected for ROI-to-ROI tests; p-values for correlations are calculated using the correlation analysis between changes in fastest 10% reaction times for PVT and functional connectivity before and after sleep deprivation.*

*^b^*p* < 0.05; Pearson’s correlation analysis*.

### Correlation Analysis Between Altered Functional Connectivity and PVT Performance

**FIGURE 4:**
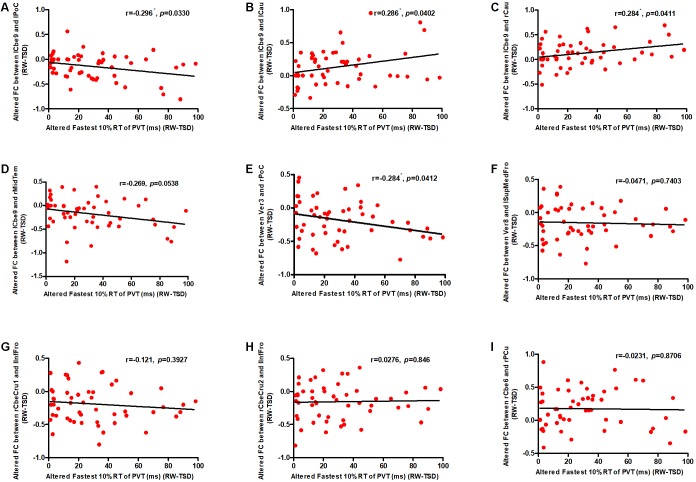
Altered functional connectivity was significantly correlated with PVT reaction times. Increase in fastest 10% reaction times were significantly negatively correlated with decrease in functional connectivity between the **(A)** lCbe9 and lPoC, and between the **(E)** Ver3 and rPoC. Increase in fastest 10% reaction times were also significantly positively correlated with decrease in functional connectivity between the **(B)** lCbe9 and lCau, and between the **(C)** lCbe9 and lCau. A negative marginally significant correlation was found between increased fastest 10% reaction times and decreased functional connectivity of **(D)** lCbe9 and rMidTem. No significant correlation was found between altered PVT indicators and functional connectivity of the **(F)** Ver8 and lSupMedFro, the **(G)** rCbeCru1 and lInfFro, the **(H)** rCbeCru2 and lInfFro, and the **(I)** rCbe6 and rPCu. ^∗^*p* < 0.05; PVT, psychomotor vigilance test; TSD, total sleep deprivation; RW, rested wakefulness; lCbe9, left cerebellum 9; lPoC, left postcentral gyrus; rCau, right caudate nucleus; lCau, left caudate nucleus; rMidTem, right middle temporal gyrus; Ver3, vermis 3; rPoC, right postcentral gyrus; Ver8, vermis 8; lSupMedFro, left superior medial frontal gyrus; rCbeCru1, right cerebellum crus 1; lInfFro, left inferior frontal gyrus; rCbeCru2, right cerebellum crus 2; rCbe6, right cerebellum 6; rPCu, right precuneus.

We conducted correlation analyses to examine the association between the increase in PVT reaction times and regions exhibiting significant changes in cerebellar functional connectivity following sleep deprivation. Our results indicated that decrease in functional connectivity between the cerebellum and the postcentral gyrus was significantly negatively correlated with increase in fastest 10% reaction times of PVT (*r*_lCbe9-lPoC_ = -0.296, *p* < 0.05; *r*_V er3-rPoC_ = -0.284, *p* < 0.05) (Figure 4A,E), while increase in functional connectivity between the cerebellum and the bilateral caudate was significantly positively correlated with increase in fastest 10% reaction times of PVT (*r*_lCbe9-lCau_ = 0.286, *p* < 0.05; *r*_lCbe9-rCau_ = 0.284, *p* < 0.05) (Figure 4B,C). Negative correlation was also found in PVT mean reaction times between the cerebellum and the postcentral gyrus (*r*_V er3-rPoC_ = -0.295, *p* < 0.05). We also found a marginally significant correlation of the functional connectivity between the left middle temporal gyrus and the left cerebellum 9 with fastest 10% reaction times of PVT (*r*_lCbe9-rMidTem_ = -0.269, *p* = 0.0538) (Figure 4D). However, no extra significant correlation were found between the changes of the indicators of PVT and alterations of functional connectivity before and after TSD (Figure 4F–I and Table 2).

## Discussion

In the present study, we investigated the effect of 36 h of sleep deprivation on the functional connectivity between the cerebellum and the cerebral cortex. We observed significant changes in functional connectivity between the cerebellum and multiple brain regions following sleep deprivation. Compared with the RW state, sleep deprivation was associated with significantly decreased functional connectivity between the cerebellar seed regions and the bilateral postcentral, left inferior frontal, left superior medial frontal, and right middle temporal gyri. In contrast, significant increase in functional connectivity was observed between the left cerebellum 9 and the bilateral caudate, and between the right cerebellum 6 and the right precuneus. We further observed that decrease in functional connectivity between the cerebellum and the postcentral gyrus was significantly correlated with increase in fastest 10% reaction times of PVT, while increase in functional connectivity between the cerebellum and the bilateral caudate was significantly correlated with increase in fastest 10% reaction times of PVT following sleep deprivation. Thus, our results indicate that acute TSD exerts a significant effect on the functional connectivity of the cerebellar network, and that such changes in connectivity are closely related to psychomotor vigilance in humans.

Strong functional connectivity was observed between the cerebellum and the bilateral caudate after sleep deprivation. While it is well-known that the cerebellum plays a key role in the regulation of movement (Bostan et al., 2010; Manto et al., 2012; Caligiore et al., 2017), research conducted during the several previous decades has revealed that it also plays an important role in the control of motor vigilance (Manto et al., 2012). Patients with cerebellar injuries may experience motor dysfunction, as well as changes in the connectivity of the motor control network (Meyer, 2016). The bilateral caudate also plays a key role in motor regulation and control of motor vigilance (Middleton and Strick, 2000; Drummond et al., 2005; Bostan et al., 2010). Therefore, the enhancement of functional connectivity between the cerebellum and the bilateral caudate may be a natural response to decrease in motor alertness after sleep deprivation. Correlation analysis revealed that these enhancements in functional connectivity were significantly positively correlated with PVT reaction times, further verifying the effect of sleep deprivation on the control of motor vigilance at the behavioral level.

In the present study, we also observed decreased functional connectivity between the cerebellum and the postcentral gyrus. The postcentral gyrus is located in the parietal lobe and primary somatosensory cortex of the human brain. The part located in the primary sensory receptive area plays an important role in the sense of touch (Iwamura et al., 1994). Recently, several studies have demonstrated that the postcentral gyrus, mainly the part located in the parietal lobe, plays an important role in motor control (Tatton et al., 1975; Porro et al., 1996; Manto et al., 2012). Our results reveal that communication between the cerebellum and the postcentral gyrus is impaired following sleep deprivation, which may indicate that motor control ability is impaired under such conditions. The PVT is regarded as a sensitive behavioral test that reflects changes in the DAN network, which is thought to play an important role in the evaluation of psychomotor vigilance (Drummond et al., 2005; Robert and Eickhoff, 2013). Decreased PVT performance is closely related to decrease in the functional connectivity between the cerebellum and the postcentral gyrus. This finding further supports the notion that parietal lobe activity and executive function decrease after sleep deprivation.

Interestingly, we also observed decreased functional connectivity between the cerebellum and the prefrontal lobe. The prefrontal lobe itself is responsible for information processing, which may not be directly involved in the control of motion alertness, but may exert its effects via interactions with the DAN network (Funahashi, 2001). Decreased functional connectivity between the cerebellum and the prefrontal lobe may indicate that alterations in the functional connections between different brain networks play an important role in impaired psychomotor vigilance following sleep deprivation. However, as we did not observe a relationship between such changes and behavior, further studies are required to determine the association between central executive control in the prefrontal lobe and the psychomotor vigilance function of the DAN.

We also found a marginally significant correlation of functional connectivity between the left middle temporal lobe and the left cerebellum 9 with PVT reaction times. This may indicate that the connection between the cerebellum and the middle temporal lobe potentially influences performance during the PVT task. However, considering the main function of the middle temporal lobe, the mechanism underlying this correlation should be studied further in the future.

In this study, we found that functional connectivity between different areas of the cerebellum and cerebral cortex changed differentially after sleep deprivation compared to before sleep deprivation. The cerebellum mainly receives information bilaterally from the cerebral cortex (especially the parietal lobe) through the pontine nucleus, via the corticopontine cerebellar pathway. The outgoing information is mainly directed to the ventrolateral thalamus and then to the anterior and primary motor cortices (Middleton and Strick, 2000; Ramnani, 2006; Caligiore et al., 2017). In this study, we found that the functional connectivity between the bilateral cerebellum and the postcentral gyrus, temporal lobe, and frontal lobe decreased significantly, while that between the caudate nucleus and the precuneus increased significantly. Further, correlation analysis showed that the changes in functional connectivity between the postcentral gyrus and the caudate nucleus were significantly correlated with the changes in PVT reaction times, which confirmed the important role of the bilateral cerebellum in the regulation of psychomotor vigilance.

The vermis also receives proprioceptive afferents related to somatic balance. There are many afferents between the vermis and the vestibular nucleus (Hirai, 1983; Barresi et al., 2012). In this study, the cerebellar vermis showed a significant decrease in functional connectivity between the frontal lobe and the postcentral gyrus. In particular, the decrease in functional connectivity between the cerebellar vermis and the postcentral gyrus was significantly correlated with the increase in PVT reaction time. These results suggest that the functions of the cerebellum related to proprioceptive processing may be impaired after sleep deprivation. Interestingly, no significant alterations were found in the functional connectivity between the thalamus and the cerebellum. It is possible that the connectivity changes mainly involve the thalamocortical pathways. The thalamocortical functional connectivity with the prefrontal and temporal cortices is altered after sleep deprivation (Shao et al., 2013), which may underlie the functional connectivity between the thalamus and the cerebellum.

The present study possesses several limitations of note. First, to simplify data collection, all the recruited volunteers were men, which may have affected the generalizability of our results. Therefore, further investigation is required to evaluate the applicability of these findings in women. Second, although behavioral monitoring was conducted to determine whether the participants were asleep, we did not obtain eye movement or electroencephalographic data to evaluate the objective depth of sleep. Third, we did not assess recovery in specific functional networks, which may provide evidence of dynamic changes in the cerebellar functional networks following sleep deprivation. Furthermore, the implications of the study findings would have been stronger if task state with PVT had been included during the scanning session. Future studies should emphasize the effect of recovery sleep on cerebellar functional connectivity following acute sleep deprivation.

In summary, the present study demonstrated that changes in functional connectivity can be observed in the brain regions associated with psychomotor vigilance after sleep deprivation. Functional connectivity between the subcortical areas (e.g., the caudate nucleus) and the cerebellum was significantly increased, that with cortical areas (e.g., the parietal and prefrontal lobes) was decreased. These findings reflect the inefficiency of the communication between the cortex and the subcortical nuclei after sleep deprivation, resulting in dysfunctional psychomotor vigilance (Houk and Wise, 1995; Middleton and Strick, 2000; Caligiore et al., 2017). The present study is the first to reveal a correlation between changes in psychomotor vigilance and changes in cerebellum function. However, further studies are required to more fully elucidate the connection between cerebellar function and psychomotor vigilance.

## Ethics Statement

This study was approved by the Research Ethics Committee of Beihang University (Beijing, China). Written informed consent was obtained from each participant after a complete description of the study had been provided. All procedures performed in studies involving human participants were in accordance with the ethical standards of the institutional and/or national research committee and with the 1964 Helsinki Declaration and its later amendments or comparable ethical standards.

## Author Contributions

YZ contributed substantially to acquisition, analysis, and interpretation of the data and drafted the article. YeY contributed substantially to analysis and interpretation of the data. YaY and JL contributed to performing the experiments and acquisition of the data. WX and YH participated in data collection and reviewed the literature. YS and XZ were the guarantors of this study, had complete access to all data in the study, and contributed substantially to conception and design as well as the interpretation of data. All authors listed have made a substantial, direct and intellectual contribution to the work, and approved it for publication.

## Conflict of Interest Statement

The authors declare that the research was conducted in the absence of any commercial or financial relationships that could be construed as a potential conflict of interest.

## Footnotes

1 http://www.fil.ion.ucl.ac.uk/spm/

2 https://www.nitrc.org/projects/conn
